# Synergistic antibacterial activity of silver nanoparticles and hydrogen peroxide

**DOI:** 10.1371/journal.pone.0220575

**Published:** 2019-08-08

**Authors:** Mahmoud Y. Alkawareek, Ahmad Bahlool, Samer R. Abulateefeh, Alaaldin M. Alkilany

**Affiliations:** School of Pharmacy, The University of Jordan, Amman, Jordan; Fondazione Pisana per la Scienza, ITALY

## Abstract

The increasing challenge of antibiotic resistance requires not only the discovery of new antibiotics, but also the development of new alternative approaches. Herein, the synergistic antibacterial activity of silver nanoparticles and hydrogen peroxide combination is reported. Unlike the bacteriostatic or slightly bactericidal activity achieved by using each agent alone, using these two agents in combination, even at relatively low concentrations, resulted in complete eradication of both the Gram negative *Escherichia coli* and the Gram positive *Staphylococcus aureus* in short treatment times indicating a clear synergistic effect between them. Modifying the surface chemistry of silver nanoparticles and the accompanied change in their surface charge enabled a further enhancement of such synergistic effect implying the importance of this aspect. Mechanistically, a Fenton-like reaction between silver nanoparticles and hydrogen peroxide is discussed and hypothesized to be the basis of the observed synergy. Achieving such a significant antibacterial activity at low concentrations reduces the potential toxicity of these agents and hence enables their utilization as an alternative antibacterial approach in wider range of applications.

## Introduction

Bacterial resistance to conventional antibiotics has become a serious problem threatening the lives of people around the globe and imposing a significant economic burden on the health sector [1,2]. The increasing challenge of this problem makes it inevitable to not only discover new antibiotics but also develop new non-antibiotic alternative approaches [3]. Among such alternatives that have received an increased attention, is the utilization of reactive oxygen species (ROS) such as hydrogen peroxide (H_2_O_2_), superoxide (O_2_^-^), ozone (O_3_), singlet oxygen (^1^O_2_), and hydroxyl radicals (OH˙); which effectively kill bacterial cells by attacking multiple cellular targets [4]. Some of these ROS, such as hydrogen peroxide, are less reactive than the others and hence relatively high concentrations of them are required to achieve sufficient activity [4]. Other ROS, such as hydroxyl radicals, are extremely reactive and exert rapid activity against bacteria even at very low concentrations [4]. However, due to very high reactivity of the latter, no stable aqueous preparations can be made of such extremely reactive species so they should be generated *in situ* (i.e. directly at their intended site of action) [5].

Several approaches have been developed for the *in situ* generation of such extremely reactive species among which is the Fenton reaction. In the conventional Fenton reaction, hydrogen peroxide reacts with ferrous ions to produce the highly bactericidal hydroxyl radicals and ferric ions [6,7]. However, since hydroxyl radicals are extremely short-lived [8,9] and ferric ions have no significant antibacterial activity, the traditional Fenton reaction is short acting and does not provide any residual activity. Furthermore, since ferrous ions, and similar metal ions, are in the dissolved state rather than particles; no surface chemistry modification is possible and hence selective targeting is not feasible with these agents which imparts high potential toxicity for the clinical utilization of this reaction. In this study, an alternative method for *in situ* generation of the bactericidal hydroxyl radicals by a Fenton-like reaction between silver nanoparticles (AgNP) and hydrogen peroxide is investigated. This method is expected to have many advantages over the conventional Fenton reaction as an alternative antibacterial approach. Among these advantages is the ability to modify the surface chemistry of silver nanoparticles by conjugation of certain moieties that enable the selective targeting of bacterial cells [10] and hence significantly reduce the toxicity on human tissues. Another advantage of the proposed approach is that, in addition to hydroxyl radicals, it produces silver ions (Ag^+^) which, not like ferric ions, have antibacterial activity themselves [11] giving the system a characteristic residual antibacterial activity. Furthermore, silver ions have been reported to also inhibit catalase enzyme [12,13] which is produced by some bacteria and neutralizes hydrogen peroxide [13] and otherwise decreases the overall activity of the system. Although the chemistry of Fenton-like reaction between silver nanoparticles and hydrogen peroxide has been recently reported in the literature [14,15], to the best of our knowledge, this is the first scientific study to investigate its utilization as an alternative antibacterial approach.

## Materials and methods

### Synthesis and characterization of silver nanoparticles

Silver nanoparticles were synthesized by the wet chemical reduction method using sodium borohydride (NaBH_4_, Sigma Aldrich, UK) as a reducing agent [16]. Polyvinyl alcohol (PVA, MW. 31–50 kDa, Sigma Aldrich, UK) and polyallylamine hydrochloride (PAH, MW. 17.5 kDa, Sigma Aldrich, UK) were used as capping/stabilizing agents for PVA-capped AgNP (PVA-AgNP) and PAH-capped AgNP (PAH-AgNP), respectively. Briefly, 1.0 mL of silver nitrate (AgNO_3_, Fisher Scientific Ltd., UK) solution (100 mM) was added to 8.0 mL of capping agent aqueous solution (1% w/v PVA or PAH) under continuous stirring at 500 rpm. To this solution, 1.0 mL of freshly prepared aqueous solution of NaBH_4_ (15 mM) was added. After that, stirring of the mixture was maintained for 30 minutes upon which a yellow/brown color developed indicating the formation of AgNP. The prepared AgNP were characterized by UV-vis absorption spectroscopy (Evolution 300 UV-Vis Spectrophotometer, Thermo Scientific, USA), transmission electron microscopy (TEM, Morgagni-Philips 268 FEI attached to MegaViewG2 Olympus Soft Imaging Solutions, USA), dynamic light scattering (DLS) and zeta potential measurements (Zetasizer Nano-ZS, Malvern instruments, UK). Average diameter of each type of the prepared AgNP was calculated from TEM images using ImageJ software.

### Kinetics of oxidative dissolution of silver nanoparticles mediated by hydrogen peroxide

In this experiment, hydrogen peroxide (H_2_O_2_, Scharlab S.L., Spain) aqueous solution (30 μL, 20 mM) was added to an aqueous dispersion of PVA-AgNP (3 mL, 0.2 mM) in a glass cuvette. UV-vis absorbance was measured at ambient conditions at 404 nm every 10 seconds for up to of 300 seconds.

### Bacterial strains and growth conditions

Bacterial strains used in this study were *Escherichia coli* ATCC 8739 and *Staphylococcus aureus* ATCC 6538. Mueller-Hinton broth (MHB, Oxoid Ltd., Basingstoke, UK) was used as a liquid growth medium while Mueller-Hinton agar (MHA, Oxoid Ltd., Basingstoke, UK) was used as a solid growth medium. Unless otherwise indicated, incubation of bacterial cultures was performed at 37°C for 18–24 hours using shaking incubator (DK-SI010, Daiki Sciences Co., Ltd., South Korea) for broth cultures and static incubator (Prebatem 2001250, J.P. SELECTA, Spain) for agar cultures. Long term bacterial stock cultures were prepared using 20% glycerin as a cryoprotectant and stored in cryogenic vials at -20°C while streak plate cultures prepared using MHA were used as short term bacterial stock cultures. Bacterial inoculum used in survival curve experiments was prepared by diluting an overnight bacterial culture in phosphate buffered saline (PBS, pH 7.4, PBS tablets, Fisher Scientific Ltd., UK) by the aid of McFarland standard to give a final density of around 1 x 10^7^ CFU/mL, which was confirmed by viable count performed by the colony count method.

### Evaluation of antibacterial activity of silver nanoparticles and hydrogen peroxide

Antibacterial activity was evaluated for PVA-AgNP and PAH-AgNP by constructing survival curves which measure the number of surviving bacterial cells over treatment time. With each type of AgNP, three different treatments were investigated including 1.0 mM AgNP alone, 10 mM H_2_O_2_ alone, and combination of 1.0 mM AgNP and 10 mM H_2_O_2_. For the combination treatment, 0.5 mL of AgNP and 0.5 mL of bacterial inoculum were added to 3.5 mL PBS in a sterile glass vial. After 15 minutes, 0.5 mL H_2_O_2_ was added to the previous mixture. Similar procedure was followed when testing the activity of each agent alone (i.e. AgNP alone and H_2_O_2_ alone) but with replacing the other agent (H_2_O_2_ and AgNP, respectively) with PBS. Furthermore, the intrinsic antibacterial activity of each capping agent (PVA or PAH) was evaluated by a procedure similar to that used for AgNP alone but with replacing the AgNP dispersion with a similar volume of the free capping agent solution. At different time points (15, 30, 45 and 60 minutes starting from the addition of H_2_O_2_), 20 μL samples were taken and immediately added to 180 μL PBS containing 0.5% w/v cysteine (L-cysteine, Sigma Aldrich, UK) as a neutralizer on which viable count was performed by the colony count method. Samples taken from a mixture of 0.5 mL bacterial inoculum and 4.5 mL PBS were used as the untreated control (i.e. to represent the zero-time point). Bacterial survival at each time point was calculated as a fraction by dividing the number of surviving cells at that point by the number of surviving cells in the corresponding control.

## Results and discussion

### Synthesis and characterization of silver nanoparticles

**Fig 1:**
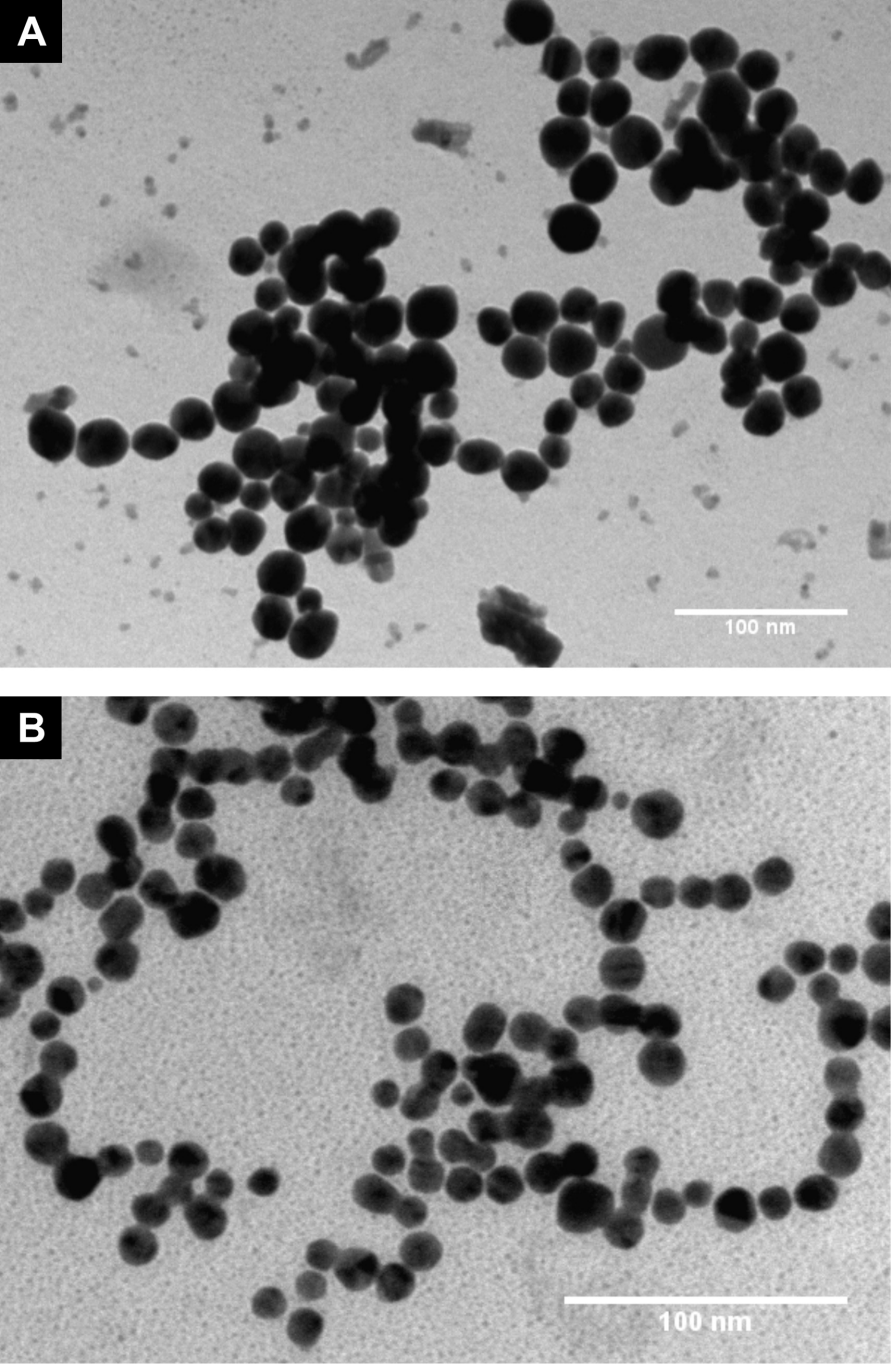
TEM images of (A) PVA-AgNP and (B) PAH-AgNP.

**Fig 2:**
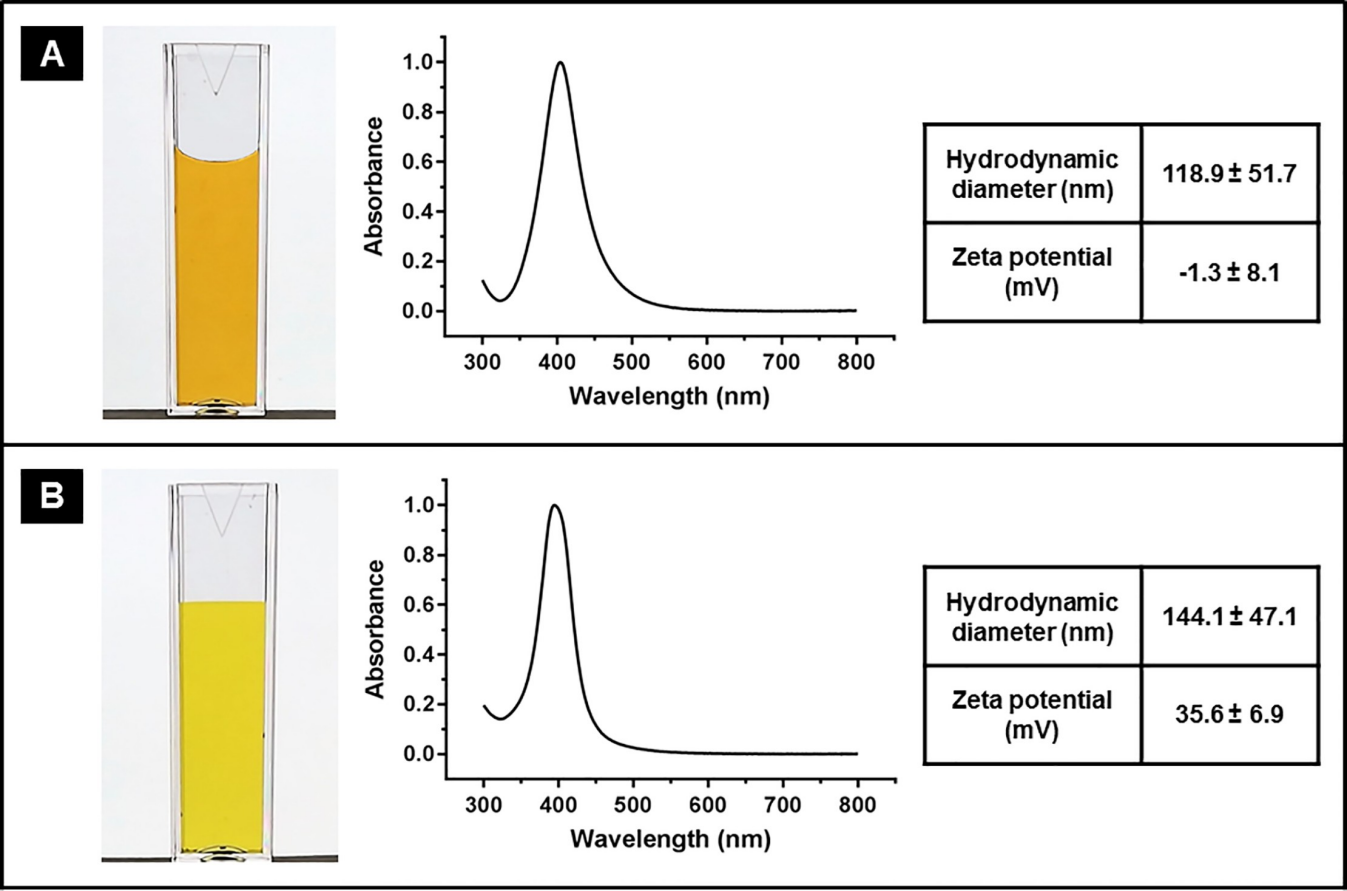
Photograph, normalized UV-vis spectrum, hydrodynamic diameter and zeta potential of (A) PVA-AgNP and (B) PAH-AgNP.

In this study, both non-ionic and cationic AgNP were prepared using two different capping agents; PVA and PAH which exhibit alcohol or amine functional groups, respectively. TEM images (presented in Fig 1) show that both types of the prepared AgNP exhibited spherical shape with average core diameter of 21.7 ± 4.7 nm for PVA-AgNP and 12.9 ± 2.3 nm for PAH-AgNP. As illustrated in Fig 2A, dispersion of PVA-AgNP exhibits a yellow/brown color with a plasmon absorption peak (λ_max_) of 404 which suggests a core diameter of around 20 nm [17,18] which is consistant to that obtained from TEM images. The narrow plasmon spectrum further indicates a homogeneous size distribution. Particle size analysis using DLS shows that the mean hydrodynamic diameter of PVA-AgNP is 118.9 nm. The significant difference between the core size of PVA-AgNP and their hydrodynamic diameter is attributed to the thickness of PVA polymer shell and the associated shell of hydration and indicates successful coating (capping) of AgNP by the polymer. Surface charge of PVA-AgNP is practically neutral as indicated by their zeta potential value of -1.3 mV confirming their non-ionic nature, which is common for PVA capped nanoparticles.

On the other hand, as shown in Fig 2B, PAH-AgNP exhibit a yellow color and their UV-vis spectrum shows a plasmon absorption peak around 397 nm which corresponds to a core diameter of around 10 nm [17,18] which is consistant to that obtained from TEM images. DLS analysis showed mean hydrodynamic diameter is 144.1 nm for PAH-AgNP; indicating the presence of PAH coating and hydration shell around AgNP. Zeta potential value of PAH-AgNP is +35.6 mV confirming the cationic nature of their surface.

### Kinetics of oxidative dissolution of silver nanoparticles mediated by hydrogen peroxide

**Fig 3:**
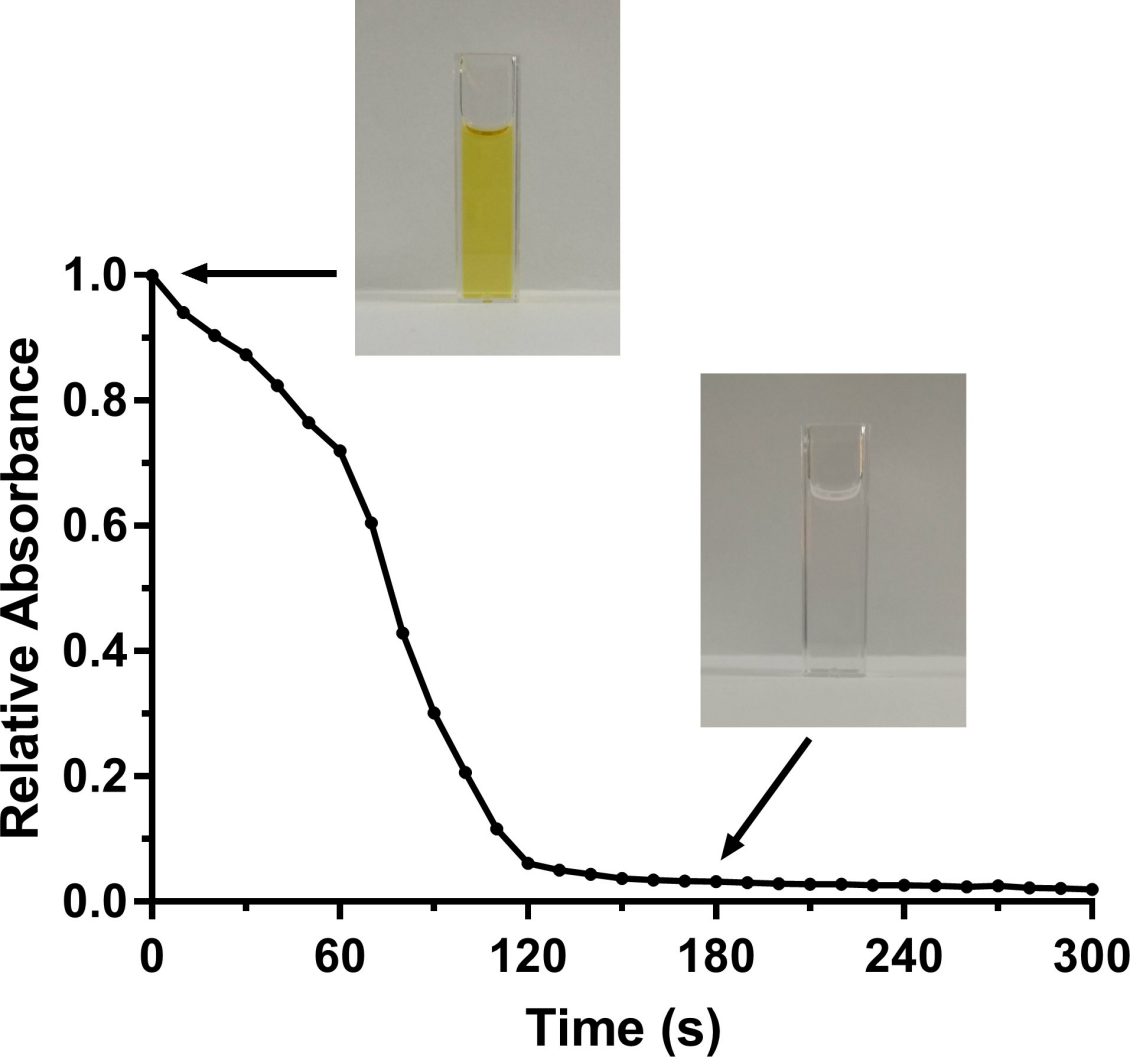
Kinetics of silver core dissolution upon H_2_O_2_ addition as measured using the relative absorbance at 404 nm. Photos above the curve show the complete disappearance of the yellowish color of AgNP dispersion upon oxidative dissolution of silver core by H_2_O_2_.

It has been reported that H_2_O_2_ undergoes a Fenton-like reaction with AgNP producing the highly reactive OH˙ and causing oxidative dissolution of AgNP into Ag^+^ [14]. A kinetic analysis of such reaction was performed in this study by following the oxidative dissolution of AgNP to Ag^+^ upon addition of H_2_O_2_. In this regard, since AgNP, but not Ag^+^, exhibit a characteristic plasmon absorption spectrum in the visible range [19], the absorbance at 404 nm was utilized to follow the dissolution of silver cores and to calculate the relative concentration of AgNP over time. As shown in Fig 3, a rapid decline in the absorbance value, indicating particle dissolution and a decline in AgNP concentration, was observed after the addition of H_2_O_2_. Such decline continued over about 3 minutes ending up with a complete dissolution of AgNP as evident from diminished absorbance and complete disappearance of initial yellowish color of AgNP dispersion.

### Synergistic antibacterial activity of silver nanoparticles and hydrogen peroxide

**Fig 4:**
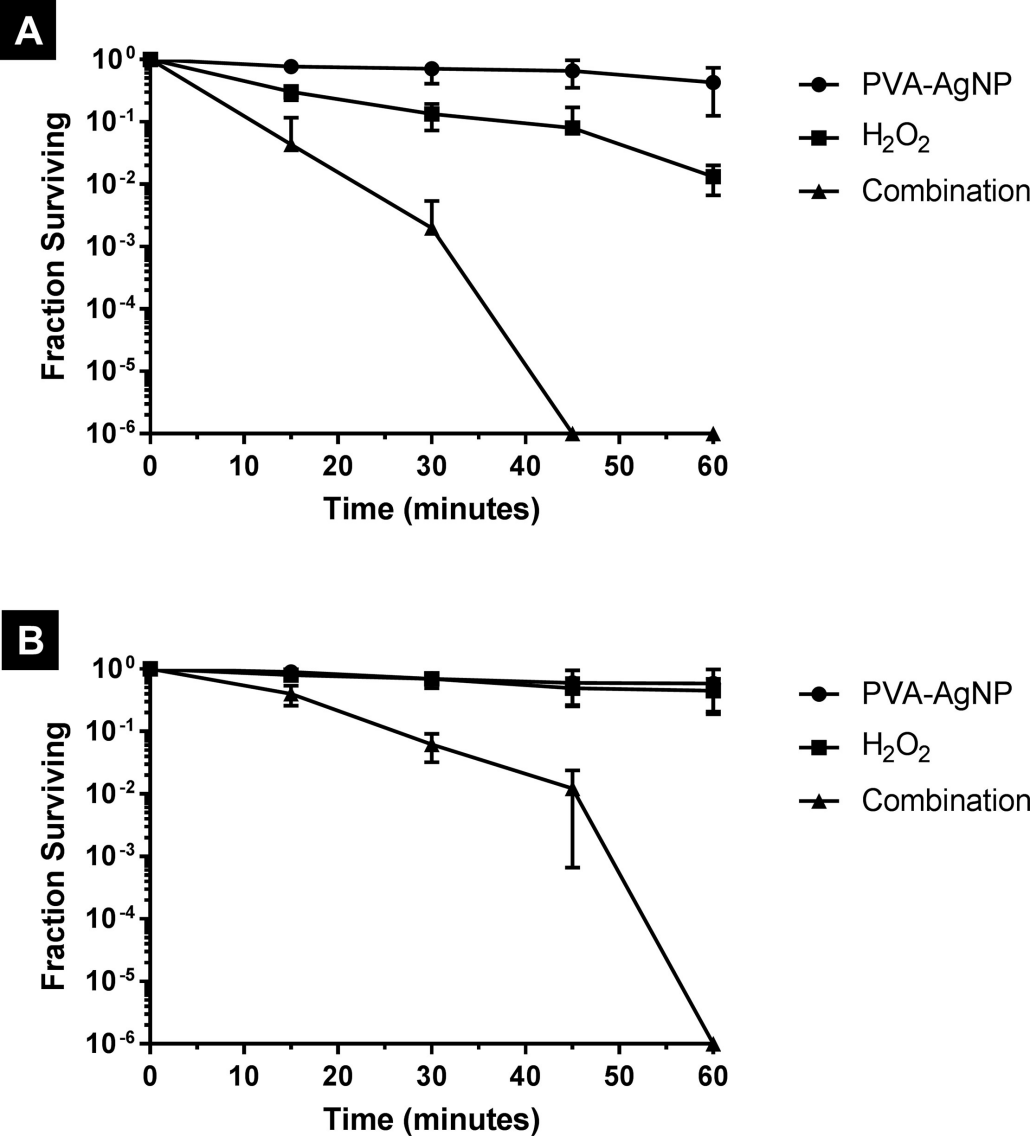
Survival curves of (A) *E*. *coli* and (B) *S*. *aureus* upon treatment with PVA-AgNP, hydrogen peroxide and their combination.

Antibacterial activity was evaluated against two different bacteria: *E*. *coli* and *S*. *aureus* to represent both Gram negative and Gram positive bacteria, respectively. Each bacteria was challenged for up to 1 hour with 3 different treatments which were: 1 mM AgNP alone, 10 mM H_2_O_2_ alone and combination of 1 mM AgNP and 10 mM H_2_O_2_. As for AgNP, two types were evaluated which are the non-ionic PVA-AgNP and the cationic PAH-AgNP. As shown in Fig 4, treatment with PVA-AgNP alone resulted in practically a bacteriostatic effect against both *E*. *coli* and *S*. *aureus* over the whole exposure time except only a slight bactericidal effect observed with *E*. *coli* after 45 minutes. On the other hand, treatment with H_2_O_2_ alone resulted in a relatively slow but continuous decrease in the viability of *E*. *coli* reaching about 2-log reduction at 60 minutes. With *S*. *aureus*, H_2_O_2_ treatment was even less effective where only a bacteriostatic effect was observed over the whole exposure time. In practice, H_2_O_2_ is commonly used at a concentration range of 3–6% (w/w) as antiseptic and general surface disinfectant and, at these concentrations, it is known to rapidly kill bacteria even at relatively short exposure times [9,20]. However, hydrogen peroxide has concentration-dependent toxicity and its use at such relatively high concentrations is associated with multiple toxic effects [21] which restrict its utilization in clinical applications. In this study the employed concentration of H_2_O_2_ is 10 mM (around 0.03% w/w); which is 100–200 times less than the usual in-use concentrations of this agent. This low concentration explains the weak bactericidal effect of H_2_O_2_ observed in this study. Interestingly, a combination of H_2_O_2_ and PVA-AgNP, even at this low concentration, resulted in a rapid decline in bacterial viability over time reaching complete killing of *E*. *coli* and *S*. *aureus* after 45 minutes and 60 minutes, respectively. With both bacterial species, the use of H_2_O_2_ and PVA-AgNP in combination resulted in increasing the bactericidal effect by more than 100 folds (i.e. more than 2-log difference) compared to either agent alone, which clearly indicates a synergistic effect between these two agents. This synergistic effect can be attributed to the Fenton-like reaction between PVA-AgNP and H_2_O_2_ that produces OH˙ [14] which is one of the most powerful biologically-active ROS [22].

**Fig 5:**
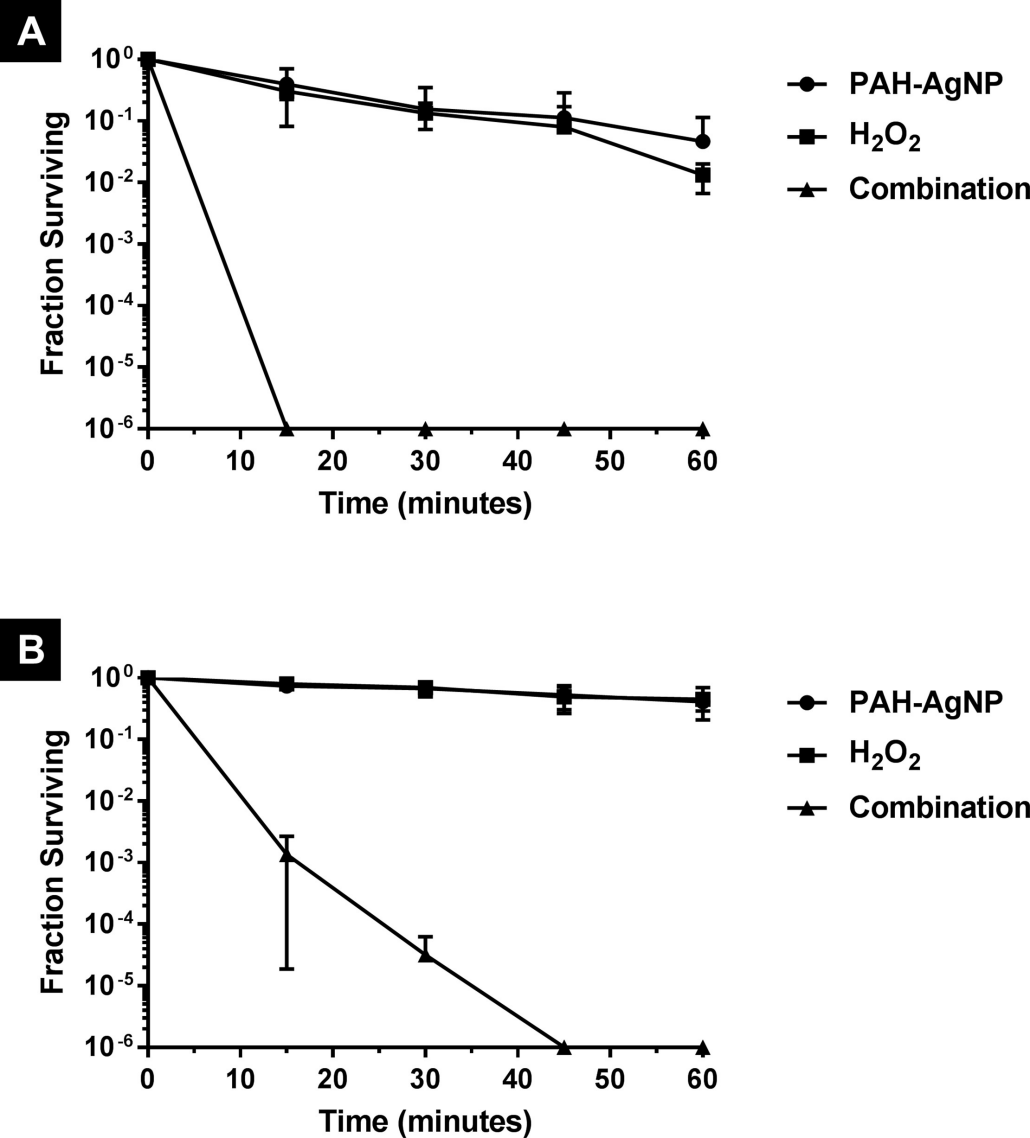
Survival curves of (A) *E*. *coli* and (B) *S*. *aureus* upon treatment with PAH-AgNP, hydrogen peroxide and their combination.

Since OH˙, which is produced by the reaction of AgNP and H_2_O_2_, is a highly reactive species and has a diffusion-limited reactivity [23]; it is not expected to travel far away from its generation site and hence can only exert its action on adjacent structures/cells. This led to a hypothesis that enhancing the adsorption of AgNP on bacterial cell surface will result in a higher amount of OH˙ being produced in the vicinity of bacterial cells and hence higher chances to react with them producing better bactericidal activity. This hypothesis was tested by performing another experiment using the cationic PAH-AgNP instead of the non-ionic PVA-AgNP. Due to their positive charge, PAH-AgNP will be more attracted (i.e. by electrostatic interaction) to the negatively charged bacterial cell surface and hence expected to adsorb in higher amounts than the non-charged PVA-AgNP [24]. As can be seen in Fig 5, the use of H_2_O_2_ in combination with PAH-AgNP successfully resulted in faster bactericidal activity, compared to that with PVA-AgNP, against both *E*. *coli S*. *aureus*. In fact, the time required by the combination to achieve complete killing was reduced from 45 to 15 minutes with *E*. *coli* and from 60 to 45 minutes with *S*. *aureus*, which can be explained and, obviously supports, the hypothesis of enhanced nanoparticle-bacteria association. However, there are other factors that may explain such an enhanced activity of the combination when using the cationic PAH as a capping agent instead of the non-ionic PVA. Among these factors are the different size [25] and the potential disruption of bacterial cell membrane that might be caused by the cationic capping agent itself [24]. Comprehensive understanding of the relative contribution of each of these factors requires further investigation and can be an interesting topic of future research. It is also worth mentioning that the activity of AgNP alone was also enhanced when using PAH, instead of PVA, as a capping agent against *E*. *coli* but not against *S*. *aureus*. However, in all cases the AgNP-H_2_O_2_ combination was much more active (more than 100 folds increase) than the activity of either AgNP alone or H_2_O_2_ alone clearly indicating a synergistic effect between AgNP and H_2_O_2_. Furthermore, control experiments evaluating the antibacterial effect of free capping agents (PVA and PAH) showed that neither one, when used alone, has a significant bactericidal activity at the concentration and exposure times used in this study (see S1 Fig in supporting information).

## Conclusions

In this study, two types of silver nanoparticles were synthesized and characterized: non-ionic PVA-capped AgNP and cationic PAH-capped AgNP. Although PVA-AgNP showed only bacteriostatic activity, their use in combination with hydrogen peroxide resulted in complete killing of both *Escherichia coli* and *Staphylococcus aureus* in 45 and 60 minutes, respectively. Treatment with PAH-AgNP alone resulted in bacteriostatic to slightly bactericidal effect but its combination with hydrogen peroxide resulted in complete killing of both *Escherichia coli* and *Staphylococcus aureus* in even shorter treatment times (15 and 45 minutes, respectively). This significantly enhanced activity of silver nanoparticles when used in combination with hydrogen peroxide clearly indicates a synergistic effect between these two agents which can be attributed to a Fenton-like reaction generating the highly bactericidal hydroxyl radicals. The high bactericidal activity of this system, its residual activity (provided by the released silver ions) and the fact that silver nanoparticles can be surface modified to allow selective targeting of bacterial cells make it possible to use this system in a much wider range of antibacterial applications compared to the traditional Fenton reaction.

## Supporting information

S1 Fig Survival curves of (A) *E. coli* and (B) *S. aureus* upon treatment with PVA and PAH polymers (i.e. free capping agents).
(TIF)
