# Supplementary figures and images for: Synergistic antibacterial activity of silver nanoparticles and hydrogen peroxide
Source: PLoS One. 2019 Aug 8;14(8):e0220575. doi: 10.1371/journal.pone.0220575 (PMC6687290; doi:10.1371/journal.pone.0220575)

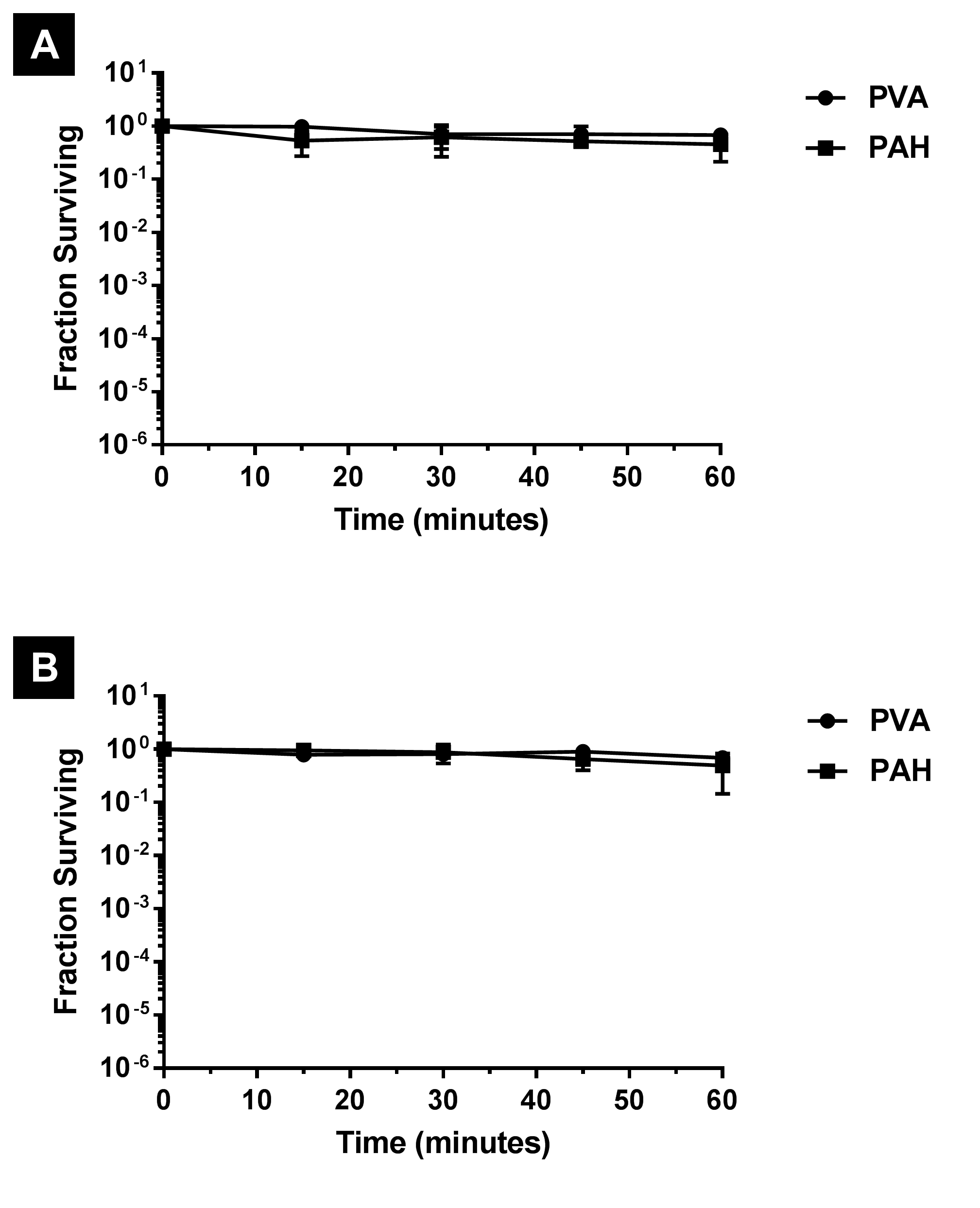

Supplement: S1 Fig — Survival curves of (A) E. coli and (B) S. aureus upon treatment with PVA and PAH polymers (i.e. free capping agents). (TIF) [file pone.0220575.s001.tif]
